# Pharmacokinetics of Caffeine following a Single Administration of Coffee Enema versus Oral Coffee Consumption in Healthy Male Subjects

**DOI:** 10.1155/2013/147238

**Published:** 2013-03-04

**Authors:** Supanimit Teekachunhatean, Nisanuch Tosri, Noppamas Rojanasthien, Somdet Srichairatanakool, Chaichan Sangdee

**Affiliations:** ^1^Department of Pharmacology, Faculty of Medicine, Chiang Mai University, Chiang Mai 50200, Thailand; ^2^Center of Thai Traditional and Complementary Medicine, Faculty of Medicine, Chiang Mai University, Chiang Mai 50200, Thailand; ^3^Phisaleeh Hospital, Nakhon Sawan 60220, Thailand; ^4^Department of Biochemistry, Faculty of Medicine, Chiang Mai University, Chiang Mai 50200, Thailand

## Abstract

The objective of this study was to determine the pharmacokinetics of caffeine after single administration of a coffee enema versus coffee consumed orally in healthy male subjects. The study design was an open-label, randomized two-phase crossover study. Eleven healthy subjects were randomly assigned either to receive 500 mL of coffee enema for 10 minutes or to consume 180 mL of ready-to-drink coffee beverage. After a washout period of at least 10 days, all the subjects were switched to receive the alternate coffee procedure. Blood samples were collected immediately before and at specific time points until 12 hours after coffee administration in each phase. The mean caffeine content in both the coffee solution prepared for the coffee enema and the ready-to-drink coffee beverage was not statistically different. The *C*_max_ and AUC of caffeine obtained from the coffee enema were about 3.5 times significantly less than those of the coffee consumed orally, despite having slightly but statistically faster *T*_max_. The *t*_1/2_ of caffeine obtained following both coffee procedures did not statistically differ. In summary, the relative bioavailability of caffeine obtained from the coffee enema was about 3.5 times significantly less than those of the coffee consumed orally.

## 1. Introduction

According to the ancient theory of “autointoxication,” the colon is believed to be a sewage system where by-products of incomplete digestion and toxins accumulate, and possibly poison the body resulting in various diseases [1–3]. Hence, some traditional physicians recommend routine treatment by the enema, a procedure involving the infusion of water or other fluids into the colon through the anus, in order to shorten the contact time of the toxins in the colon [4, 5]. A coffee enema is one of the ancient medical procedures still in use today for “detoxification” since Dr. Max Gerson introduced it for the purpose of cancer therapy in the 1930s. According to the Gerson regimen [5, 6], caffeine from the coffee enema is believed to cause dialysis of toxic products from blood across the colonic walls or to cause dilation of the bile ducts, which in turn facilitates the process of elimination of toxic products from the liver. Nonetheless, none of these claims regarding the production of substantial health benefits by coffee enema and other colonic cleansing treatments has been supported by scientific research [7–9]. Although the evidence for health benefits is lacking, the usage of coffee enema is still very popular among patients in Thailand, especially in the treatment of cancers, allergies, asthma, urticaria, migraine, dyslipidemia, obesity and chronic constipation, and so forth.

The documented potential risks of coffee enema include rectal burn induced by hot enema fluid [10, 11], proctocolitis [12, 13], polymicrobial enteric septicemia [14], electrolyte imbalance, or even death [15]. In addition, systemic adverse effects from colonic absorption of caffeine following a coffee enema are another issue that might be of concern to enema users or even mainstream physicians. In fact, several lines of evidence suggest that caffeine can be absorbed via the rectum or colon into systemic circulation as it has been used as a test drug in the evaluation of pressure-controlled colon delivery capsules (PCDCs) [16]. Furthermore, caffeine can be used in combination with ergotamine in rectal suppository dosage form in order to enhance the vasoconstrictive effect of ergotamine in the treatment of migraine headache [17]. However, the colonic absorption of caffeine following coffee enema considerably differs from that following administration of a PCDC or a rectal suppository because the coffee enema procedure usually involves administration of a larger volume of warm and diluted coffee solution (about 500 mL) into the rectum and colon through the anus. Additionally, the subject is normally requested to retain the coffee enema fluid for a short period of time (about 10–15 min) before defecation, leading to limitation of duration of caffeine absorption. Based on this, it is hypothesized that the extent of caffeine absorption should be rather low, especially in relation to that following an administration of PCDC, rectal formulation, or even oral coffee consumption. Despite this, no scientific research regarding caffeine pharmacokinetics following coffee enema has yet been reported. The primary objective of this study was therefore to compare the pharmacokinetic parameters of caffeine after a single dose of the coffee enema with those of coffee which was consumed orally in healthy male subjects. The secondary objective was to evaluate the safety profile of a single administration of these coffee procedures on blood pressure and heart rate.

## 2. Materials and Methods

### 2.1. Study Design

The present study was a substudy related to the investigation which has been reported elsewhere by Teekachunhatean et al. [9]. This study was an open-label, randomized two-phase crossover study. Eleven healthy subjects were randomly assigned to receive either 500 mL of coffee enema for 10 minutes or to consume 180 mL of coffee beverage. After a washout period of at least 10 days, all subjects were switched to receive the alternate coffee procedure. Subjects were randomized by a computer-generated list. The allocation sequence was implemented through placing the allocation cards in opaque, sealed, and stapled envelopes to preserve concealment. The envelopes were numbered in advance and opened sequentially only after the enrolled subjects completed all baseline assessments, and it was time to allocate which coffee procedure should be administered first in a given sequence.

### 2.2. Subjects

A total of 11 healthy men, aged 18–25 years (y), were enrolled in this study. The body mass index of each subject had to be within 18–25 kg/m^2^. All had to be in good health on the basis of medical history and a physical examination. Routine blood tests including a complete blood count, a liver function test and a measurement of blood urea nitrogen and creatinine levels were used to screen subjects in order to exclude those with abnormal hematological diseases or abnormal liver or kidney functions. All subjects had to have normal blood pressure and heart rate. During the screening phase, subjects had to be able to retain the water enema for at least 10 min. Subjects included in the study were given thorough verbal and written information regarding the nature of the study. Signed informed consent of each subject was obtained prior to the study. Exclusion criteria included subjects who were not able to avoid foods or drinks that contained caffeine within the previous 10 days and during the study period, as well as those with a known history of any gastrointestinal disease such as peptic ulcer, hemorrhoids, gut obstruction, diverticulitis, ulcerative colitis, Crohn's disease, irritable bowel syndrome (IBS), colostomy, recent bowel surgery, and colorectal cancer. Other exclusion criteria were chronic renal, liver, neurological, pulmonary, or cardiovascular diseases, recent cigarette smoking within the previous 3 months, a history of substance abuse or addiction, the use of any medication within the previous month, and hypersensitivity to medications in the xanthine group such as theophylline or aminophylline. Withdrawal criteria of this study were those subjects who experienced adverse drug reactions during the study, subjects who could not comply with the study protocol or those who wished to voluntarily withdraw from the study, and any subject who required other medication during the study period.

### 2.3. Coffee Enema and Coffee Enema Procedure

The coffee solution used in the enema procedure was prepared by mixing 4 g of finely ground coffee beans (VS coffee, manufactured by V.S. coffee, Thailand) with 100 mL of purified water. The solution was boiled at 100°C for 15 minutes (min) and then simmered at 60°C for approximately 15 min. Afterwards, the solution was filtered using a fine sieve, adjusting the total volume to 500 mL, and it was then allowed to cool to 37°C.

The coffee enema devices used in this study were the disposable commercial set (Cleansing Enema set, made in Mexico, imported by Thanyaphu Co. Ltd., Thailand) consisting of a plastic nozzle connected by a tube to a plastic bag which would contain the coffee enema fluid. The nozzle was lubricated with 2 drops of organic olive oil and then inserted 2 inches into the anus while the subject was lying down on his left side, with his legs curled into the abdomen. The bed height was 3 feet above the floor, whereas the enema bag was hung 5 feet above the floor. The coffee solution in the enema bag was completely infused within 5–10 min. The subject was requested to retain the coffee enema fluid for 10 min. During this period, the subject was instructed to change his lying position to the right side for 3.5 min and then switched back to the left side for 3.5 min and finally to the supine position for 3 min before evacuation. The enema procedure was performed at the Clinical Pharmacology Unit, Department of Pharmacology, Faculty of Medicine, Chiang Mai University, with the assistance of the research nurse. 

### 2.4. Oral Coffee Consumption

The coffee used for oral consumption in this study was the commercially available ready-to-drink coffee beverage (instant coffee with milk and sugar), Red Bull Coffee manufactured by TC Pharmaceutical Industry Co., Ltd. The net volume of 1 serving was 180 mL. Each subject was instructed to consume the entire coffee serving within 1 min followed by 100 mL of water.

### 2.5. Administration of Coffee Enema and Oral Coffee Consumption

Subjects were requested to visit the Clinical Pharmacology Unit, Department of Pharmacology, Faculty of Medicine, Chiang Mai University on the days specified according to the protocol schedule. They were randomly assigned to receive either the coffee enema or to orally consume the coffee beverage. Blood samples were collected at different specific time points (see below). After blood sample collection 12 hours (h) after dose, the subjects were discharged from the Clinical Pharmacology Unit. After a washout period of at least 10 days, subjects were switched to the second phase receiving the alternate coffee preparation, and the blood samples were collected in the same manner. Identical meals and fluids were served during the 2 study phases. All subjects were required to refrain from drinking beverages containing caffeine (except those given in this study) and alcohol from the time of screening until the end of the research study. After initiating each coffee procedure, subjects continued fasting until water and lunch were served 2 h and 6 h afterwards, respectively.

### 2.6. Blood Sample Collection for Determination of Caffeine Pharmacokinetic Parameters

In each study phase, subjects were fasted overnight for at least 8 h. Venous blood samples were taken via heparinized IV catheter inserted into a forearm vein. Fifteen mL of blood samples were drawn from each subject prior to an administration of either the coffee enema or oral coffee consumption and again 10, 20, 30, 40, and 60 min and 1.5, 2, 4, 8, and 12 h after each procedure. The blood collecting tubes were centrifuged at 1,200 rpm for 15 min, and the plasma was separated and frozen at −80°C for later analysis.

### 2.7. Determination of Caffeine Concentrations in Coffee Solutions

The assay of caffeine content was modified from the high-performance liquid chromatography (HPLC) method and conditions previously reported elsewhere [18, 19]. One mL of each coffee preparation (either enema solution or ready-to-drink coffee beverage) was diluted 10-fold with 10% methanol and was then spiked with 10 *μ*L of internal standard (IS, 100 *μ*g/mL acetaminophen). Five *µ*L of sample solution was injected into the HPLC system. Chromatographic separation was performed on 5 *μ*m C_18_, 100 × 4.6 i.d. analytical and guard columns. The chromatography condition consisted of two mobile phases. The mobile phase A used was 1 mmol/L perchloric acid/isopropanol (1,000/56, v/v)/2.2 mmol/L sodium dodecyl sulfate which was pumped through the column at a flow rate of 1 mL/min for 7 min. The mobile phase B used was 1 mmol/L perchloric acid/isopropanol (1,000/88, v/v)/3 mmol/L sodium dodecyl sulfate and was pumped through the column at a flow rate of 1 mL/min for 8 min, and the analytes were detected by UV absorption at 274 nm, while the column was maintained at 40°C. The caffeine content of the unknown samples was determined using a calibration curve of peak height ratios of caffeine and IS versus respective caffeine concentrations (2,500–100,000 ng/mL) with the use of linear regression.

### 2.8. Determination of Caffeine Concentrations in Plasma

**Figure 1:**
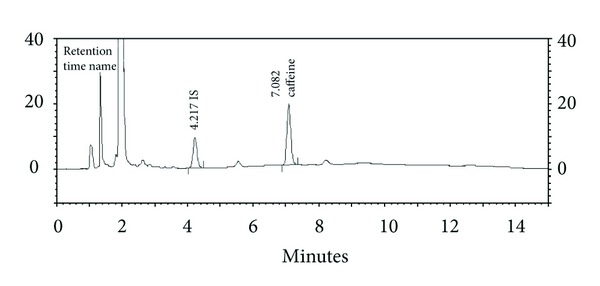
Chromatogram of plasma sample containing 4.00 *μ*g/mL of internal standard (IS, retention time = 4.217 min) and 4.00 *μ*g/mL of caffeine (retention time = 7.082 min).

The assay was modified from the protein precipitation procedure previously described elsewhere [18, 20]. Two hundred and fifty *μ*L of sample plasma was spiked with 10 *μ*L of IS and then deproteinated by mixing the plasma sample with 380 *μ*L of acetonitrile and kept at room temperature for 20 min. After vortex mixing, the protein was removed by centrifugation at 14,000 g (room temperature) for 5 min. An aliquot of the supernatant (600 *μ*L) was removed and evaporated to be vacuum dried for 2 h at 60°C. The residue was reconstituted with 50 *μ*L of mobile phase B and then vortex spun for 20 seconds. Five *μ*L of this solution was injected into the HPLC system as described above. Chromatogram of plasma containing caffeine and IS is presented in Figure 1. Plasma concentrations of caffeine were determined by interpolating the peak height ratios of caffeine and IS versus respective caffeine concentrations (0.1–4 *μ*g/mL).

The percentage of coefficient of variation (%CV) of intraday precision for plasma caffeine concentrations ranged from 1.69 to 3.91%, whereas, the %CV of interday precision ranged from 4.47 to 5.78%. The deviation of intraday and interday assay for plasma caffeine concentrations ranged from −8.45 to 2.00% and −3.43 to 5.52%, respectively. The lower limit of quantification (LLOQ) was 0.1 *μ*g/mL. The mean recovery of caffeine from the determination procedure was 96.97%.

### 2.9. Determination of Blood Pressure and Heart Rate

Blood pressure and heart rate were measured using Omron digital blood pressure monitor (IntelliSense, Model HEM-711, Omron Healthcare, Inc.) prior to either the coffee enema or the oral coffee consumption and again at 10, 20, 30, 40, and 60 min and 1.5, 2, 4, 8, and 12 h following both coffee procedures. Subjects were instructed to maintain a relaxed, semirecumbent position for a 5-min stabilization period before each measurement. 

### 2.10. Data Analysis and Statistical Methods

All statistical analyses were performed using the SPSS package for Windows and StatsDirect 2.5.6. All data were compared with two-side test. Differences were considered statistically significant at *P* < 0.05.

The primary outcomes of this study were the pharmacokinetics parameters of caffeine, which were the maximal plasma concentration (*C*_max⁡_), the time to the maximal plasma concentration (*T*_max⁡_), the area under the plasma concentration-time curve from time 0 to 12 h (AUC_0–12_) and from time 0 to infinity (AUC_0–∞_), and the half-life (*t*_1/2_). The *C*_max⁡_ and *T*_max⁡_ were obtained directly by visual inspection of each subject's plasma concentration time profile. The AUC_0–12_, AUC_0–∞_ and *t*_1/2_ were determined by noncompartmental analysis using the TopFit software version 2.0 for personal computer. The slope of the terminal log-linear portion of the concentration-time curve was determined by least-squares regression analysis and was used for the calculation of the elimination rate constant (*k*_*e*_). The elimination *t*_1/2_ was calculated as 0.693/*k*_*e*_. The AUC_0–12_ was calculated using the trapezoidal rule. Extrapolated AUC from time *t* to infinity (AUC_*t*–*∞*_) was determined as *C*_*t*_/*k*_*e*_. Total AUC was the sum of AUC_0–12_ + AUC_12–∞_. 

The pharmacokinetic parameters were presented as mean ± SD. The differences of the mean values of *C*_max⁡_, AUC_0–12_, AUC_0–∞_, *T*_max⁡_, and *t*_1/2_ between both coffee procedures were statistically analyzed using paired *t*-test. Additionally, the 95% CIs were calculated in order to analyze associations between different procedures. The differences of the mean values of plasma caffeine concentrations at any specific time points between both coffee procedures were also analyzed using paired *t*-test.

The secondary outcomes were the hemodynamic parameters after administration of each caffeine procedure. The mean values of systolic and diastolic blood pressure, and heart rate between baseline and at any specific time points after initiation of each coffee procedure were compared using one-way ANOVA with repeated measurement.

## 3. Results

Eleven healthy Thai male subjects were enrolled in the study. Their mean values of age, weight, height, and BMI were 21.09 ± 7.97 yr, 58.86 ± 9.58 kg, 1.68 ± 0.07 m, and 20.80 ± 2.27 kg/m^2^, respectively. The mean values of systolic blood pressure, diastolic blood pressure, and heart rate (HR) were 112.40 ± 6.87 mm Hg, 73.00 ± 8.52 mm Hg, and 69.20 ± 12.62 beat/min, respectively.

Six servings of each coffee solution were measured for caffeine content. The mean caffeine contents were 107.24 ± 2.22 mg/500 mL for coffee enema solution and 96.34 ± 1.39 mg/180 mL for ready-to-drink coffee beverage. These mean values of the caffeine contents were not statistically different between the coffee solution prepared for the enema and the ready-to-drink coffee beverage (*P* = 0.972).

**Figure 2:**
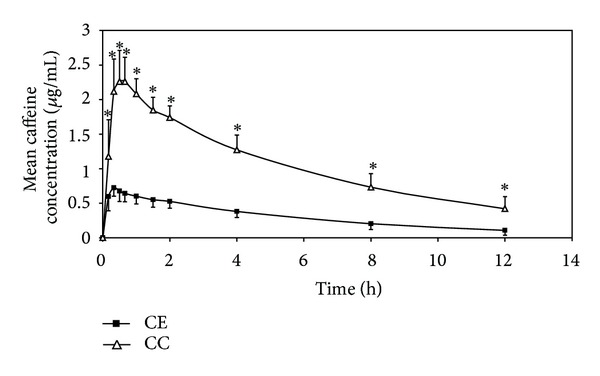
Mean plasma caffeine concentration-time curves following a single administration of the coffee enema (CE) or the oral coffee consumption (CC) (*n* = 11). *Statistically significant between groups (*P* < 0.05, paired *t*-test).

The mean plasma caffeine concentration-time profiles and the pharmacokinetic parameters of caffeine (*C*_max⁡_, AUC_0–12_, AUC_0–∞_, *T*_max⁡_, and *t*_1/2_) after a single administration of the coffee enema and the oral coffee consumption are shown in Figure 2 and Table 1, respectively. The mean values of *C*_max⁡_, AUC_0–12_, and AUC_0–∞_ of caffeine obtained from the coffee enema was about 3.5 times significantly less than those of the coffee consumed orally, despite having slightly but statistically faster *T*_max⁡_. Nonetheless, the mean *t*_1/2_ of caffeine obtained following both coffee procedures did not statistically differ.

A single administration of either the coffee enema or the coffee drink produced no statistical change in systolic blood pressure, diastolic blood pressure, and heart rate when compared to their own baseline values (data not shown). 

## 4. Discussion

In the present study, “unfiltered boiled coffee” (boiled coffee without filtration through fine-paper filter) was chosen for the preparation of the enema fluid according to the instruction established by Gerson and because it contains relatively high levels of bioactive compounds with antioxidant activity [6]. The dose of coffee edema (4 g of finely ground coffee beans), the volume of the enema fluid (500 mL), and the retaining duration (10 min) were chosen according to commonly practiced habit in the Thai coffee enema users. In contrast, since “instant coffee with milk and sugar” seems to be the most popular type of coffee drink in Thailand, the “ready-to-drink” beverage of this instant coffee was therefore chosen for the coffee consumption in this study because the amount of caffeine in each serving is consistent and has been standardized by the manufacturing company. Since the caffeine contents in both coffee enema fluid and ready-to-drink coffee beverage were not statistically different, it could be assumed that the pharmacokinetic study of caffeine following a single dose of both coffee procedures was conducted using the comparable dose of caffeine. 

It has been demonstrated that caffeine absorption from the gastrointestinal tract, especially in the small intestine, is rapid and complete with the bioavailability of 99 to 100% after oral administration [21–27]. *T*_max⁡_ can be as wide as 15 to 120 min because of variations in gastric emptying [27–29]. Our results demonstrated that oral administration of a single dose of the coffee drink in fasting condition resulted in rapid absorption of caffeine with the average *T*_max⁡_ of 0.44 h (26.4 min). When the *C*_max⁡_ of oral caffeine absorption in our study was compared to those reported in other studies, our study demonstrated that a single administration of coffee consumption, containing 96.34 mg of caffeine, resulted in the average *C*_max⁡_ of 2.47 *μ*g/mL comparable to *C*_max⁡_ of 1.5–1.8 *μ*g/mL following a single 100 mg oral dose of caffeine [30, 31]. 

Caffeine can be also used in combination with some medications in suppository dosage form [17]. This data lends support to the notion that caffeine can be absorbed into systemic circulation when administered via the rectum or colon. It has been demonstrated that a single administration of rectal formulation of indomethacin/prochlorperazine/caffeine containing 75 mg of caffeine in adult patients with migraine and episodic tension-type headache exhibited averaged *C*_max⁡_ of 5.2 *μ*g/mL and averaged *T*_max⁡_ of 1.9 h [32]. In contrast, the present study revealed that a single administration of 500 mL of coffee enema fluid containing a comparable content of caffeine (107.24 mg) resulted in a remarkably lower extent (*C*_max⁡_ and AUC) but faster *T*_max⁡_ (0.30 h or 18 min) of absorbed caffeine. This discrepancy in pharmacokinetic parameters of caffeine following coffee enema versus caffeine rectal suppository might result from the possibility that the coffee enema fluid was retained for only 10 min, and the unabsorbable caffeine was then emptied from the large intestine by defecation afterwards. This factor limits the time for caffeine absorption into systemic circulation via the large intestine [33]. This might be a theoretical possibility which could also be used to explain why the coffee enema resulted in a significantly lower extent (*C*_max⁡_ and AUC) and faster *T*_max⁡_ of absorbed caffeine compared with the orally consumed coffee, when the comparable doses of caffeine were administered in this study. 

Nonetheless, the mean plasma *t*_1/2_ of caffeine derived from the coffee enema or the orally consumed coffee (4.68 versus 4.87 h) did not significantly differ. Additionally, these values were also comparable to those of 2.5–5.7 h reported in other studies investigating the *t*_1/2_ of caffeine after oral administration [21, 34]. This similarity is a result of the fact that caffeine is eliminated by first-order kinetics. With first order elimination, the elimination rate constant is independent of plasma concentration and routes of administration.

 This study showed that a single dose of coffee orally consumed containing 96.34 mg/serving of caffeine exerted no statistical changes in the systolic blood pressure, diastolic blood pressure, and heart rate when compared to the baseline values. These data are consistent with the results reported in previous studies. Indeed, several lines of evidence reported no change in these hemodynamic parameters after approximately 100–200 mg of caffeine (either in the form of coffee or purified caffeine) when administered orally to healthy subjects [9, 35–37]. Since the coffee enema resulted in a significantly lower extent of caffeine absorption, it is therefore not surprising that the coffee enema did not produce statistically significant clinical changes in such hemodynamic parameters when compared to the baseline values. These findings confirm that a single administration of coffee enema, with a given coffee concentration and volume mentioned in the present study, should not produce deleterious effects on the hemodynamics in healthy subjects. Nevertheless, since most of the caffeine in systemic circulation is normally eliminated within 4-5 half-lives (approximately 24 h) [38], it could be postulated that even multiple doses of coffee enemas (e.g., once a day or once every other day) would not result in the accumulation of caffeine in the body and hence should not adversely affect hemodynamic parameters. This postulation is in accordance with the results from our previous study demonstrating that multiple doses of a coffee enema (3 times weekly for 6 visits) do not adversely affect either the hemodynamic parameters or the electrolyte balance in healthy male subjects [9]. However, since caffeine is known to be extensively metabolized by hepatic cytochrome P-450 1A2 (CYP1A2) [39], and this metabolizing enzyme appears to be polymorphically distributed in human populations; therefore, CYP1A2 slow metabolizers possibly exhibit higher plasma caffeine concentrations and more pronounced hemodynamic effects following the coffee enema than the rapid metabolizers. This is likely to be an issue that warrants further investigation.

Although our previous study has demonstrated that single or multiple doses of coffee enema do not produce beneficial effects with respect to an enhancement of serum glutathione levels and trolox equivalent antioxidant capacity or a decrease in serum malondialdehyde concentrations in the same population reported here [9], documented evidence exists that it might be associated with considerable potential risks. It is worth noting that such procedure should be performed by trained and skillful personnel using appropriate equipment in subjects or patients without contraindication (i.e., colorectal cancer, recent bowel surgery, colostomy, gut obstruction, hemorrhoids, diverticulitis, Crohn's disease, ulcerative colitis, irritable bowel syndrome, etc.).

Some important limitations need to be considered regarding the present study. Firstly, the coffee enema fluid of which its mean caffeine content and the total volume were restricted to 107.24 mg/500 mL. In addition, each subject was requested to retain the coffee enema fluid for exactly 10 min while lying in various postures before defecation. In a real situation, coffee enema users might perform this procedure using different types of coffee with wide variation in coffee concentrations/volumes as well as retaining durations. The variations in these factors might influence colonic absorption and pharmacokinetics of caffeine following enema procedure and warrant further investigation. Secondly, this study compared the caffeine pharmacokinetics using the different types of coffee (unfiltered boiled coffee for enema *versus *ready-to-drink coffee for consumption) according to commonly practiced habits in the Thai population, further study investigating the same type of coffee liquid (e.g., unfiltered boiled coffee) with the same concentration and comparable dose should be encouraged. Thirdly, this investigation was conducted using a small sample size (*n* = 11). However, this small sample size was still able to demonstrate the statistical difference in caffeine pharmacokinetics between both coffee procedures, suggesting that the sample size should be considered adequate. Fourthly, this study was preliminarily investigated in male subjects. The female subjects were excluded in order to rule out the effects from sex hormone fluctuations during the menstrual cycles which might confound the volume of distribution and other pharmacokinetic parameters of caffeine between the two study phases [40]. Nonetheless, since there are no gender differences in pharmacokinetics of caffeine [41], the findings in male subjects reported here might be generalized to both male and female adults. Finally, slow and rapid phenotypes of caffeine metabolizing enzymes (e.g., CYP1A2, acetyltransferase, etc.) in the enrolled subjects should be screened prior to study participation, and pharmacokinetic study of caffeine following coffee enema in the different phenotypes warrants further investigation. 

## 5. Conclusions

When comparable content of caffeine was administered, the *C*_max⁡_ and AUC of caffeine obtained from the coffee enema were about 3.5 times significantly less than those of the coffee consumed orally, despite having slightly but statistically faster *T*_max⁡_. In addition, a single administration of the coffee enema or the oral coffee consumption did not adversely affect systolic and diastolic blood pressure and heart rate.

## Figures and Tables

**Table 1:** Pharmacokinetic parameters of caffeine following a single administration of the coffee enema or the oral coffee consumption (*n* = 11).

Parameters	Coffee procedure				95% confidence interval of the difference		*P* value*
	Coffee enema	Oral coffee consumption	Mean difference	Standard error difference	Lower	Upper
*C*_max⁡_ (*μ*g/mL)	0.77 ± 0.14	2.47 ± 0.39	−1.70	0.12	−1.96	−1.44	<0.001
AUC_0–12_ (*μ*g·h/mL)	3.69 ± 0.95	13.05 ± 2.06	−9.36	0.71	−10.83	−7.89	<0.001
AUC_0–*∞*_ (*μ*g·h/mL)	4.73 ± 1.74	16.32 ± 3.89	−11.59	1.28	−14.27	−8.84	<0.001
*T*_max⁡_ (h)	0.30 ± 0.12	0.44 ± 0.11	−0.14	0.05	−0.24	−0.02	0.02
*t*_1/2_ (h)	4.68 ± 1.36	4.87 ± 1.39	−0.19	0.59	−1.41	1.04	0.76

Data represents mean ± SD. Abbreviations: *C*_max⁡_: maximum plasma caffeine concentrations, AUC_0–12_: area under the concentration-time curve from administration to 12 hours, AUC_0–*∞*_: area under the concentration-time curve from administration and extrapolation to infinity, *T*_max⁡_: time to reach maximum plasma concentration, and *t*_1/2_: half-life. *Statistical analysis using paired Student's *t*-test.
